# 2,4,5-Tri-2-furyl-1*H*-imidazole

**DOI:** 10.1107/S1600536809049514

**Published:** 2009-11-25

**Authors:** Shuai-Jun Wang, Qiang Gu, Qing Su, Xiao-Dong Chen, Yu-Min Zhang

**Affiliations:** a College of Chemistry, Jilin University, Changchun 130012, People’s Republic of China; b Experimental Center of Testing Science, Jilin University, Changchun 130023, People’s Republic of China

## Abstract

In the crystal of the title compound, C_15_H_10_N_2_O_3_, the molecules are linked together by inter­molecular N—H⋯N hydrogen bonds into chains along the *c* axis. The crystal structure also shows weak inter­molecular C—H⋯π hydrogen bonds. The three furanyl rings bonded to the imidazole core are not coplanar with the latter; the dihedral angles between the furanyl and imidazole ring planes are 29.3 (2), 19.4 (2), and 4.8 (2)°.

## Related literature

For background to imidazole derivatives, see: Ho *et al.* (2003 ▶); Lambardino *et al.* (1974 ▶); Bao *et al.* (2003 ▶); Fürstner *et al.* (2000 ▶); Sundberg *et al.* (1996 ▶).
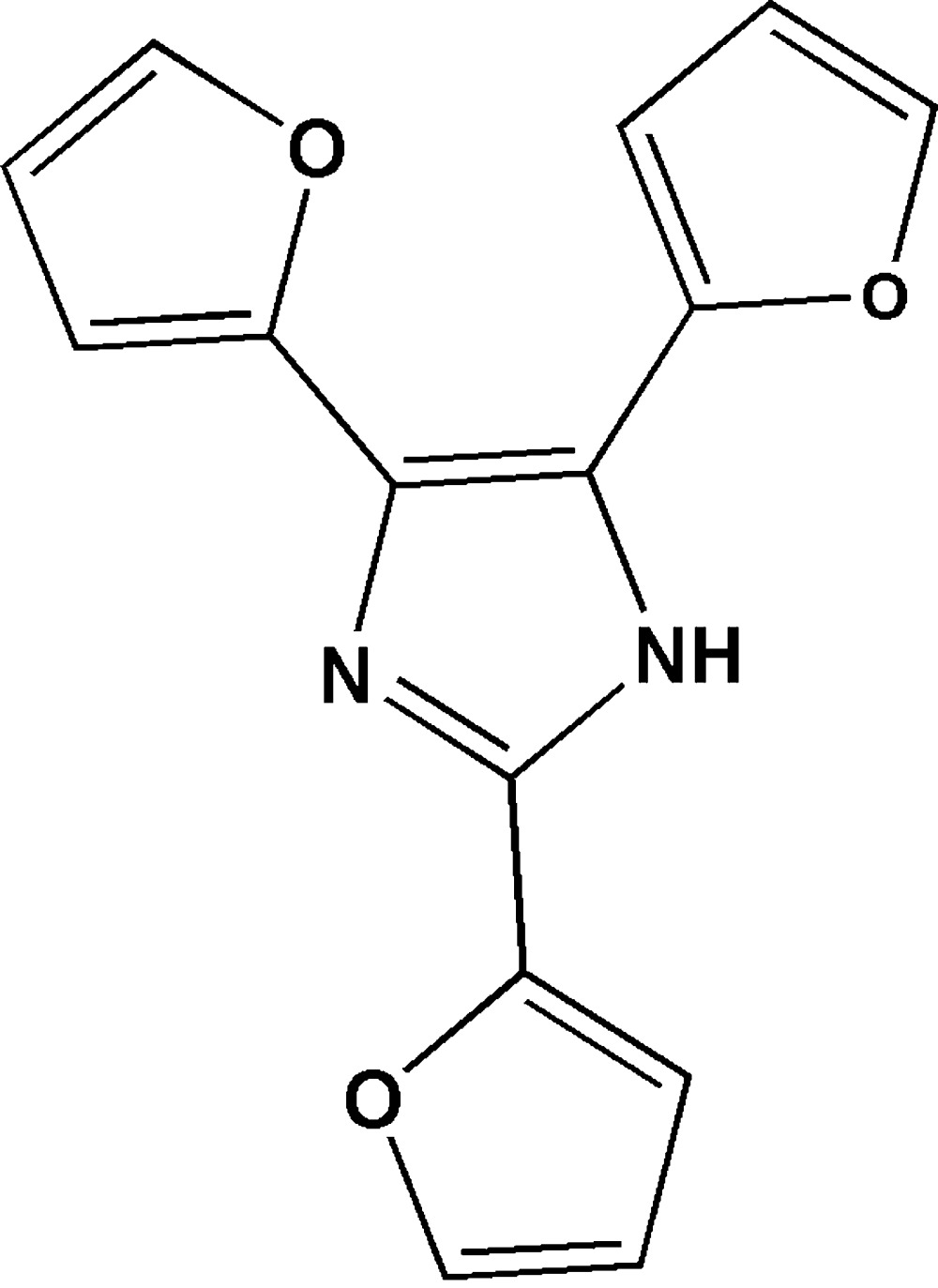


## Experimental

### 

#### Crystal data

C_15_H_10_N_2_O_3_
*M* *_r_* = 266.25
Monoclinic, 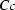

*a* = 9.3940 (19) Å
*b* = 17.146 (3) Å
*c* = 9.1484 (18) Å
β = 116.29 (3)°
*V* = 1321.1 (5) Å^3^
*Z* = 4
Mo *K*α radiation
μ = 0.10 mm^−1^
*T* = 295 K
0.26 × 0.24 × 0.12 mm

#### Data collection

Rigaku R-AXIS RAPID diffractometer
Absorption correction: multi-scan (*SADABS*; Bruker, 2001 ▶) *T*_min_ = 0.975, *T*_max_ = 0.989
6430 measured reflections
1514 independent reflections
1089 reflections with *I* > 2σ(*I*)
*R*_int_ = 0.031

#### Refinement

*R*[*F* ^2^ > 2σ(*F* ^2^)] = 0.042
*wR*(*F* ^2^) = 0.119
*S* = 1.04
1514 reflections
185 parameters
2 restraints
H atoms treated by a mixture of independent and constrained refinement
Δρ_max_ = 0.13 e Å^−3^
Δρ_min_ = −0.14 e Å^−3^

### 

Data collection: *RAPID-AUTO* (Rigaku Corporation, 1998 ▶); cell refinement: *RAPID-AUTO*; data reduction: *CrystalStructure* (Rigaku/MSC, 2002 ▶); program(s) used to solve structure: *SHELXS97* (Sheldrick, 2008 ▶); program(s) used to refine structure: *SHELXL97* (Sheldrick, 2008 ▶); molecular graphics: *XP* in *SHELXTL* (Sheldrick, 2008 ▶); software used to prepare material for publication: *SHELXL97*.

## Supplementary Material

Crystal structure: contains datablocks global, I. DOI: 10.1107/S1600536809049514/om2298sup1.cif

Structure factors: contains datablocks I. DOI: 10.1107/S1600536809049514/om2298Isup2.hkl

Additional supplementary materials:  crystallographic information; 3D view; checkCIF report

## Figures and Tables

**Table 1:** Hydrogen-bond geometry (Å, °)

*D*—H⋯*A*	*D*—H	H⋯*A*	*D*⋯*A*	*D*—H⋯*A*
N1—H1*A*⋯N2^i^	0.97 (3)	1.94 (3)	2.899 (3)	167 (2)
C10—H10⋯*Cg* ^ii^	0.93	2.81	3.6031 (31)	144

Symmetry codes: (i) 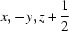; (ii) 
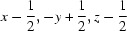
. *Cg* is the centroid of the N1/C5/C6/N2/C11 ring.

**Fig. 1.**
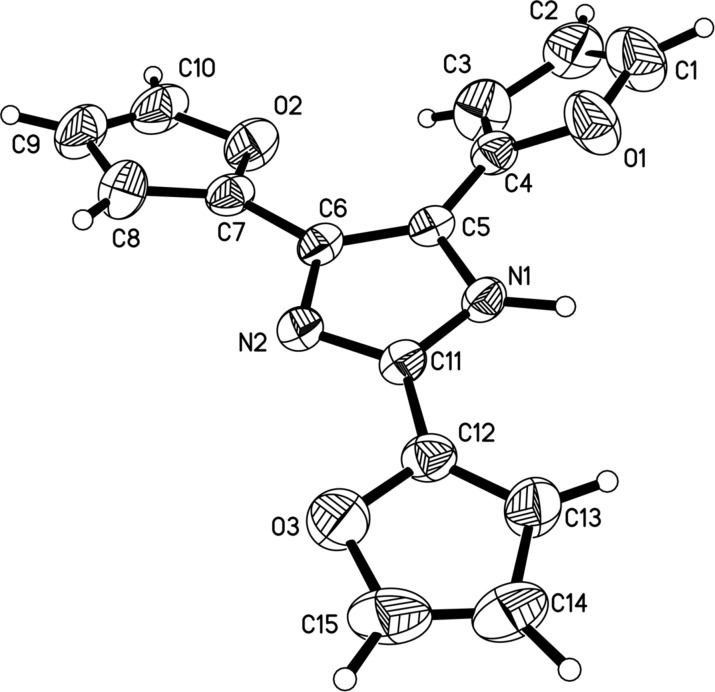
The molecule of the title compound, showing the atom labelling scheme. Displacement ellipsoids are drawn at the 30% probability level and H atoms are shown as small spheres of arbitrary radii.

**Fig. 2.**
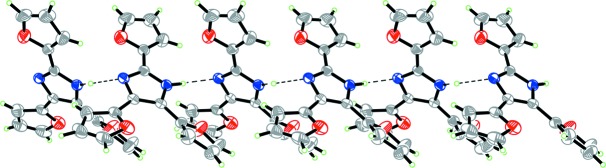
Packing of the molecules showing the intermolecular N—H···N interactions to form a 1-D chain. Color scheme: blue, nitrogen; red, oxygen; grey, carbon; green, hydrogen.

**Fig. 3.**
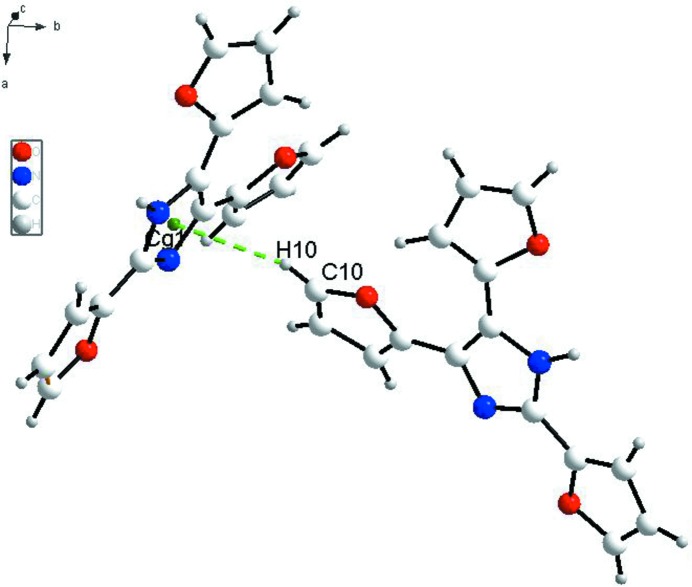
Packing of the molecules showing the C—H···π interactions. Cg is the centroid of the imidazole ring (N1/C5/C6/N2/C11). C10···Cg = 3.6031 (31) Å. The green spots are the centers of the aromatic rings.

**Fig. 4.**
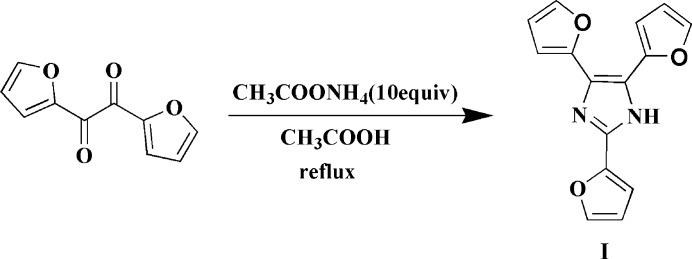
The formation of the title compound.

## supplementary crystallographic information

### Comment

As an important member of the five-membered heterocycles, the imidazole moiety is present in a wide range of naturally occurring molecules (Ho *et al.*, 2003). Compounds with an imidazole ring system have many pharmacological properties and play important roles in biochemical processes. (Lambardino & Wiseman, 1974). The biological importance of the imdazole ring system has made it a common substructure in numerous synthetic compounds such as fungicides, herbicides, plant growth regulators and therapeutic agents. Recent advances in green chemistry and organometallic chemistry have extended the boundary of imidazoles to the synthesis and application of a large class of imidazoles as ionic liquids (Bao *et al.*, 2003) and imidazole-related N-heterocyclic carbenes (Fürstner *et al.*, 2000). Compounds containing the furan ring easily react with singlet oxygen (Sundberg *et al.*, 1996). As most devices are operated under an oxygen-free environment, in recent years furan derivatives have been used in research of photoelectric materials.

In the crystal structure of the title molecule (Fig.1), the three furan rings and the imidazole ring are not coplanar. The dihedral angles between the three furan rings C1/C2/C3/C4/O1, C7/C8/C9/C10/O2, C12/C13/C14/C15/O3 and the imidazole ring N1/C5/C6/N2/C11 are 29.3, 19.4 and 4.8°, respectively. The neighbouring molecules are nearly vertical to each other with the dihedral angle 98.0° and linked together by intermolecular N—H···N hydrogen bonds into 1-D infinite chains along the *c* axis (Table 1, Fig.2). The crystal structure also shows weak intermolecular C—H···π hydrogen bonds (Fig. 3), Cg = centroid of N1/C5/C6/N2/C11.

### Experimental

A mixture of furil (5.26 mmol, 1 g) and ammonium acetate (52.6 mmol, 4.05 g) in acetic acid (20 ml) was refluxed. After completion of the reaction confirmed by TLC, the reaction mixture was cooled to room temperature, poured into 100 ml of water, and then neutralized with a 20% NaOH aqueous solution to pH 9. The mixture was extracted with ethyl acetate, and the solvent was removed by rotary evaporation. The crude product was further purified by column chromatography using a mixture of petroleum ether and ethyl acetate (3:1) as eluents. Then 2,4,5-tri(furan-2-yl)-1*H*-imidazole was recystallized from methanol. Yellow single crystals were obtained by slow evaporation of the solvent at ambient temperature. For C_15_H_10_N_2_O_3_, MS: 267.2 (*M*+H)^+1^, found:266.2.

### Refinement

The C-bound H atoms were positioned geometrically with C—H = 0.93 Å, and allowed to ride on their parent atoms in the riding model approximation with *U*_iso_(H) = 1.2 *U*_eq_(C). The H atom attached to N was found in a difference Fourier map and refined isotropically. Friedel opposites were merged.

### Crystal data

**Table:**

C_15_H_10_N_2_O_3_	*Z* = 4
*M**_r_* = 266.25	*F*(000) = 552
Monoclinic, *C**c*	*D*_x_ = 1.339 Mg m^−^^3^
Hall symbol: C -2yc	Mo *K*α radiation, λ = 0.71073 Å
*a* = 9.3940 (19) Å	θ = 3.4–27.5°
*b* = 17.146 (3) Å	µ = 0.10 mm^−^^1^
*c* = 9.1484 (18) Å	*T* = 295 K
β = 116.29 (3)°	Block, yellow
*V* = 1321.1 (5) Å^3^	0.26 × 0.24 × 0.12 mm

### Data collection

**Table:**

Rigaku R-AXIS RAPID diffractometer	1514 independent reflections
Radiation source: fine-focus sealed tube	1089 reflections with *I* > 2σ(*I*)
graphite	*R*_int_ = 0.031
ω scans	θ_max_ = 27.5°, θ_min_ = 3.4°
Absorption correction: multi-scan (*SADABS*; Bruker, 2001)	*h* = −12→12
*T*_min_ = 0.975, *T*_max_ = 0.989	*k* = −22→22
6430 measured reflections	*l* = −11→10

### Refinement

**Table:**

Refinement on *F*^2^	Primary atom site location: structure-invariant direct methods
Least-squares matrix: full	Secondary atom site location: difference Fourier map
*R*[*F*^2^ > 2σ(*F*^2^)] = 0.042	Hydrogen site location: inferred from neighbouring sites
*wR*(*F*^2^) = 0.119	H atoms treated by a mixture of independent and constrained refinement
*S* = 1.04	*w* = 1/[σ^2^(*F*_o_^2^) + (0.0752*P*)^2^] where *P* = (*F*_o_^2^ + 2*F*_c_^2^)/3
1514 reflections	(Δ/σ)_max_ < 0.001
185 parameters	Δρ_max_ = 0.13 e Å^−^^3^
2 restraints	Δρ_min_ = −0.14 e Å^−^^3^

### Special details

**Table:**

Experimental. (See detailed section in the paper)
Geometry. All e.s.d.'s (except the e.s.d. in the dihedral angle between two l.s. planes) are estimated using the full covariance matrix. The cell e.s.d.'s are taken into account individually in the estimation of e.s.d.'s in distances, angles and torsion angles; correlations between e.s.d.'s in cell parameters are only used when they are defined by crystal symmetry. An approximate (isotropic) treatment of cell e.s.d.'s is used for estimating e.s.d.'s involving l.s. planes.
Refinement. Refinement of *F*^2^ against ALL reflections. The weighted *R*-factor *wR* and goodness of fit *S* are based on *F*^2^, conventional *R*-factors *R* are based on *F*, with *F* set to zero for negative *F*^2^. The threshold expression of *F*^2^ > σ(*F*^2^) is used only for calculating *R*-factors(gt) *etc*. and is not relevant to the choice of reflections for refinement. *R*-factors based on *F*^2^ are statistically about twice as large as those based on *F*, and *R*- factors based on ALL data will be even larger.

### Fractional atomic coordinates and isotropic or equivalent isotropic displacement parameters (Å^2^)

**Table:**

	*x*	*y*	*z*	*U*_iso_*/*U*_eq_	
O1	0.2149 (4)	0.03841 (17)	0.1765 (4)	0.0969 (10)	
O2	0.2353 (3)	0.22150 (13)	−0.1681 (3)	0.0785 (7)	
O3	0.7630 (4)	−0.05039 (19)	−0.0235 (3)	0.0887 (8)	
N1	0.4736 (3)	0.01826 (14)	0.1105 (3)	0.0514 (6)	
H1A	0.484 (5)	−0.016 (2)	0.199 (5)	0.071 (10)*	
N2	0.5145 (3)	0.06255 (15)	−0.0952 (3)	0.0523 (6)	
C1	0.1128 (7)	0.0677 (3)	0.2310 (8)	0.1106 (17)	
H1	0.0680	0.0389	0.2862	0.133*	
C2	0.0868 (6)	0.1396 (3)	0.1967 (6)	0.0962 (14)	
H2	0.0198	0.1716	0.2203	0.115*	
C3	0.1802 (6)	0.1624 (3)	0.1147 (5)	0.0918 (13)	
H3	0.1872	0.2118	0.0767	0.110*	
C4	0.2547 (4)	0.09777 (18)	0.1044 (4)	0.0558 (7)	
C5	0.3664 (3)	0.07872 (16)	0.0416 (3)	0.0488 (6)	
C6	0.3936 (3)	0.10452 (16)	−0.0868 (3)	0.0499 (7)	
C7	0.3183 (3)	0.16580 (17)	−0.2043 (4)	0.0564 (7)	
C8	0.3121 (5)	0.1808 (2)	−0.3509 (4)	0.0832 (11)	
H8	0.3586	0.1518	−0.4043	0.100*	
C9	0.2190 (5)	0.2505 (3)	−0.4089 (6)	0.0884 (13)	
H9	0.1935	0.2754	−0.5077	0.106*	
C10	0.1770 (5)	0.2724 (2)	−0.2969 (6)	0.0882 (14)	
H10	0.1162	0.3162	−0.3035	0.106*	
C11	0.5600 (3)	0.01121 (16)	0.0265 (3)	0.0492 (6)	
C12	0.6862 (4)	−0.04443 (19)	0.0694 (4)	0.0588 (8)	
C13	0.7492 (5)	−0.0938 (2)	0.1934 (5)	0.0807 (12)	
H13	0.7166	−0.1013	0.2747	0.097*	
C14	0.8742 (5)	−0.1329 (3)	0.1795 (7)	0.0974 (14)	
H14	0.9409	−0.1704	0.2501	0.117*	
C15	0.8781 (6)	−0.1061 (3)	0.0482 (7)	0.1003 (15)	
H15	0.9489	−0.1224	0.0086	0.120*	

### Atomic displacement parameters (Å^2^)

**Table:**

	*U*^11^	*U*^22^	*U*^33^	*U*^12^	*U*^13^	*U*^23^
O1	0.102 (2)	0.0895 (19)	0.136 (3)	0.0189 (17)	0.085 (2)	0.0234 (17)
O2	0.0788 (15)	0.0619 (14)	0.0816 (15)	0.0195 (13)	0.0236 (12)	0.0141 (12)
O3	0.0850 (18)	0.111 (2)	0.0780 (15)	0.0260 (16)	0.0436 (14)	0.0038 (15)
N1	0.0502 (13)	0.0540 (13)	0.0454 (12)	0.0098 (11)	0.0170 (10)	0.0059 (10)
N2	0.0473 (12)	0.0571 (14)	0.0476 (12)	−0.0017 (12)	0.0165 (10)	−0.0001 (10)
C1	0.104 (4)	0.124 (4)	0.142 (4)	0.027 (3)	0.089 (4)	0.017 (3)
C2	0.084 (3)	0.118 (4)	0.098 (3)	0.045 (3)	0.051 (2)	0.009 (3)
C3	0.108 (3)	0.077 (2)	0.095 (3)	0.029 (2)	0.049 (2)	0.002 (2)
C4	0.0501 (16)	0.0563 (17)	0.0575 (16)	0.0085 (14)	0.0208 (13)	0.0027 (13)
C5	0.0430 (13)	0.0484 (14)	0.0467 (14)	0.0027 (12)	0.0123 (10)	−0.0007 (11)
C6	0.0469 (14)	0.0453 (15)	0.0464 (14)	−0.0003 (12)	0.0107 (11)	−0.0005 (11)
C7	0.0481 (16)	0.0486 (15)	0.0588 (16)	−0.0060 (13)	0.0111 (12)	0.0037 (12)
C8	0.093 (3)	0.079 (2)	0.072 (2)	0.010 (2)	0.032 (2)	0.0216 (19)
C9	0.084 (3)	0.078 (2)	0.079 (2)	0.001 (2)	0.014 (2)	0.035 (2)
C10	0.073 (2)	0.067 (2)	0.096 (3)	0.0108 (19)	0.011 (2)	0.030 (2)
C11	0.0473 (14)	0.0504 (15)	0.0454 (13)	0.0050 (13)	0.0164 (11)	−0.0010 (12)
C12	0.0528 (17)	0.0630 (18)	0.0578 (16)	0.0080 (14)	0.0222 (14)	−0.0051 (14)
C13	0.086 (3)	0.078 (2)	0.087 (2)	0.037 (2)	0.047 (2)	0.026 (2)
C14	0.080 (3)	0.087 (3)	0.111 (3)	0.039 (2)	0.029 (2)	0.016 (3)
C15	0.072 (2)	0.111 (3)	0.117 (4)	0.031 (3)	0.042 (3)	−0.018 (3)

### Geometric parameters (Å, °)

**Table:**

O1—C4	1.352 (4)	C4—C5	1.440 (4)
O1—C1	1.358 (5)	C5—C6	1.381 (4)
O2—C7	1.363 (4)	C6—C7	1.444 (4)
O2—C10	1.370 (4)	C7—C8	1.341 (5)
O3—C12	1.340 (4)	C8—C9	1.437 (6)
O3—C15	1.373 (6)	C8—H8	0.9300
N1—C11	1.349 (4)	C9—C10	1.306 (7)
N1—C5	1.387 (4)	C9—H9	0.9300
N1—H1A	0.96 (4)	C10—H10	0.9300
N2—C11	1.332 (4)	C11—C12	1.434 (4)
N2—C6	1.375 (4)	C12—C13	1.326 (5)
C1—C2	1.269 (7)	C13—C14	1.406 (6)
C1—H1	0.9300	C13—H13	0.9300
C2—C3	1.438 (7)	C14—C15	1.302 (7)
C2—H2	0.9300	C14—H14	0.9300
C3—C4	1.336 (5)	C15—H15	0.9300
C3—H3	0.9300		
			
C4—O1—C1	107.1 (3)	C8—C7—C6	132.3 (3)
C7—O2—C10	106.9 (3)	O2—C7—C6	118.2 (3)
C12—O3—C15	106.3 (4)	C7—C8—C9	106.2 (4)
C11—N1—C5	107.9 (2)	C7—C8—H8	126.9
C11—N1—H1A	124 (2)	C9—C8—H8	126.9
C5—N1—H1A	128 (2)	C10—C9—C8	107.2 (3)
C11—N2—C6	105.5 (2)	C10—C9—H9	126.4
C2—C1—O1	111.0 (4)	C8—C9—H9	126.4
C2—C1—H1	124.5	C9—C10—O2	110.3 (4)
O1—C1—H1	124.5	C9—C10—H10	124.8
C1—C2—C3	107.4 (4)	O2—C10—H10	124.8
C1—C2—H2	126.3	N2—C11—N1	111.4 (2)
C3—C2—H2	126.3	N2—C11—C12	126.1 (3)
C4—C3—C2	105.7 (4)	N1—C11—C12	122.5 (2)
C4—C3—H3	127.1	C13—C12—O3	109.4 (3)
C2—C3—H3	127.1	C13—C12—C11	131.3 (3)
C3—C4—O1	108.9 (3)	O3—C12—C11	119.3 (3)
C3—C4—C5	135.5 (3)	C12—C13—C14	107.5 (4)
O1—C4—C5	115.6 (3)	C12—C13—H13	126.2
C6—C5—N1	104.8 (3)	C14—C13—H13	126.2
C6—C5—C4	135.2 (3)	C15—C14—C13	106.3 (4)
N1—C5—C4	119.9 (3)	C15—C14—H14	126.8
N2—C6—C5	110.4 (2)	C13—C14—H14	126.8
N2—C6—C7	118.9 (3)	C14—C15—O3	110.4 (4)
C5—C6—C7	130.7 (3)	C14—C15—H15	124.8
C8—C7—O2	109.4 (3)	O3—C15—H15	124.8

### Hydrogen-bond geometry (Å, °)

**Table:**

*D*—H···*A*	*D*—H	H···*A*	*D*···*A*	*D*—H···*A*
N1—H1A···N2^i^	0.97 (3)	1.94 (3)	2.899 (3)	167 (2)
C10—H10···Cg^ii^	0.93	2.81 (1)	3.6031 (31)	144 (1)

Symmetry codes: (i) *x*, −*y*, *z*+1/2; (ii) *x*−1/2, −*y*+1/2, *z*−1/2.
